# Functional Scaffolding for Brain Implants: Engineered Neuronal Network by Microfabrication and iPSC Technology

**DOI:** 10.3389/fnins.2019.00890

**Published:** 2019-08-29

**Authors:** Kenta Shimba, Chih-Hsiang Chang, Takahiro Asahina, Fumika Moriya, Kiyoshi Kotani, Yasuhiko Jimbo, Arseniy Gladkov, Oksana Antipova, Yana Pigareva, Vladimir Kolpakov, Irina Mukhina, Victor Kazantsev, Alexey Pimashkin

**Affiliations:** ^1^Department of Precision Engineering, School of Engineering, The University of Tokyo, Tokyo, Japan; ^2^Department of Neuroengineering, Center of Translational Technologies, N. I. Lobachevsky State University of Nizhny Novgorod, Nizhny Novgorod, Russia; ^3^Department of Molecular and Cellular Technologies, Central Research Laboratory, Privolzhsky Research Medical University, Nizhny Novgorod, Russia; ^4^Department of Neurotechnology, N. I. Lobachevsky State University of Nizhny Novgorod, Nizhny Novgorod, Russia

**Keywords:** human induced pluripotent stem cell, microelectrode array, microfabrication, neuronal network, 3D scaffold brain implant, neural tissue engineering, microfluidics

## Abstract

Neuroengineering methods can be effectively used in the design of new approaches to treat central nervous system and brain injury caused by neurotrauma, ischemia, or neurodegenerative disorders. During the last decade, significant results were achieved in the field of implant (scaffold) development using various biocompatible and biodegradable materials carrying neuronal cells for implantation into the injury site of the brain to repair its function. Neurons derived from animal or human induced pluripotent stem (iPS) cells are expected to be an ideal cell source, and induction methods for specific cell types have been actively studied to improve efficacy and specificity. A critical goal of neuro-regeneration is structural and functional restoration of the injury site. The target treatment area has heterogeneous and complex network topology with various types of cells that need to be restored with similar neuronal network structure to recover correct functionality. However, current scaffold-based technology for brain implants operates with homogeneous neuronal cell distribution, which limits recovery in the damaged area of the brain and prevents a return to fully functional biological tissue. In this study, we present a neuroengineering concept for designing a neural circuit with a pre-defined unidirectional network architecture that provides a balance of excitation/inhibition in the scaffold to form tissue similar to that in the injured area using various types of iPS cells. Such tissue will mimic the surrounding niche in the injured site and will morphologically and topologically integrate into the brain, recovering lost function.

## Introduction

In the field of regenerative medicine, neural tissue regeneration can be performed with implantation of 3D scaffold structures containing progenitor cells. Such structures composed of biodegradable materials (polymers, hydrogels, and hyaluronic acid) provide integration of cells in the central nervous system (CNS) and the brain with defined cellular density, dissolving after a few days and leaving only the cells in the site of injury (Ovsianikov et al., 2011; Wang et al., 2012; Akopova et al., 2015; Carlson et al., 2016; Jin et al., 2016; Timashev et al., 2016; Venugopalan et al., 2016; Balyabin et al., 2017; Chen et al., 2018).

While identifying optimal biodegradable materials for scaffold is an ongoing problem, several key problems remain unresolved with respect to neural tissue integration as well.

**FIGURE 1:**
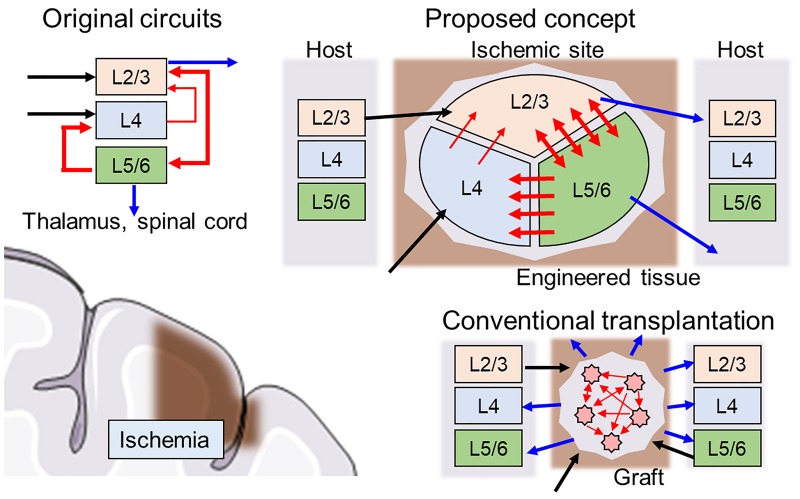
Schematics of hybrid neural network transplantation. Cerebral cortex has a unique layered structure, and the layers are interconnected as shown in the left upper panel. Blue, black, and red arrows indicate input, output, and intra-cortical connections, respectively. In conventional transplantation using cell suspension, the intra-cortical connections and output to neighboring tissue usually are randomly distributed and not ordered. In proposed method, the engineered tissue with biologically natural and heterogeneous structure is transplanted to damaged area. The cell components are made by specific induction of pluripotent stem cells. The directionality of synaptic connectivity formed by microfabricated structures.

First, the direction of the neurite outgrowth after transplantation must be controlled to facilitate integration into signaling pathways of the host brain tissue. Thus, the network architecture should be heterogeneous to replace the injured site with similar architecture (connectome). For example, a cortical column is organized as a multilayered structure where interlayer connectivity is organized with unidirectional synaptic connections, and each layer consists of various types of neurons present at a certain proportion (Figure 1, upper left). When the cerebral cortex experiences ischemia (Figure 1, lower left), conventional transplantation is not sufficient to restore the original network because homogeneous cell populations cannot form specific network structures (Figure 1, lower right).

Second, the neural tissue should consist of appropriate cell populations similar to the original tissue to recovery of function. One of the most perspective approaches is to use induced pluripotent stem (iPS) cells or stem cells with scaffolds (see review by Medvedeva et al., 2018). This approach recently was successfully applied in a transplantation to human retina without forming a tumor for over a year (Mandai et al., 2017). Excitatory and inhibitory neurons are the most common cells and should be studied first.

Recent advances in microfluidics, soft lithography, and material science have resulted in the development of new technologies to address these issues by using dissociated neurons or iPS cells. Neuronal cultures have been demonstrated to exhibit basic *in vivo*-like phenomena, such as information encoding and transfer in networks (Gal et al., 2010), network synaptic plasticity (Shahaf and Marom, 2001), and memory (Dranias et al., 2013). We propose a new type of heterogeneous structure of the scaffold, in which various types of cells form biologically realistic networks with unidirectional synaptic connectivity mimic injured brain regions with functional integration, thereby potentially recovering cognitive behavior (Figure 1).

## Concept

A key feature of the proposed scaffold is an inner geometric structure that determines network architecture development and is compatible with the host brain structure (Figure 1, upper right). The example in Figure 1 shows a schematic design of the scaffold with three separate clusters of neurons of various types connected through asymmetric microchannels (red arrows) to drive a specific direction of neurite outgrowth during integration in the brain. After several days, connectivity is formed between the clusters, and the scaffold degrades, leaving only newly formed and structured tissue.

**FIGURE 2:**
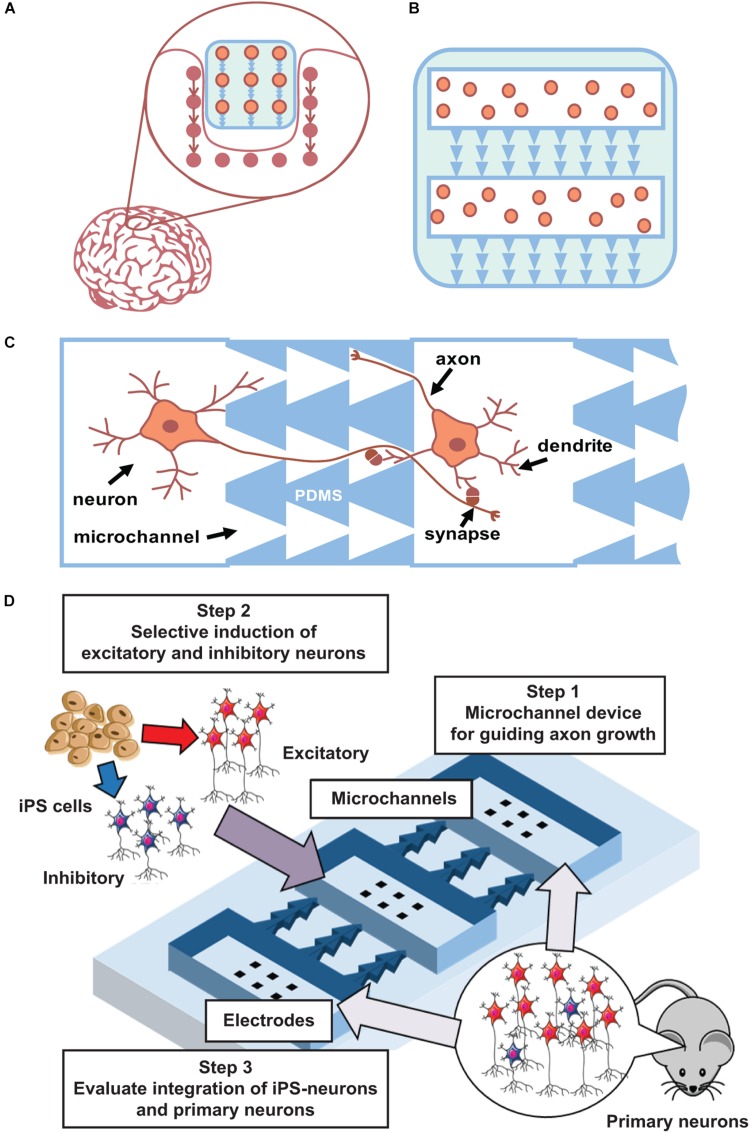
Main concept. **(A)** Concept of integration a “friendly” network. **(B)** Scaffold with iPS cells and asymmetric microchannels integrates to similar surrounding network structure. 2D case can be done using current state microfluidics with PDMS chips. **(C)**. Schematic view of microchannels (PDMS) that couples two neuronal cultures and provide unidirectional axon growth in between. **(D)** Scheme of iPS cells integration to developed network in a microfluidic chip.

The first steps of such method development can be performed using planar neuronal cultures grown in multi-chamber microfluidic devices. Such devices contain several chambers for cell plating that are connected by microchannels of asymmetric shape to promote unidirectional axonal growth between cultures (Figure 2) and to promote the formation of synapses with pre-defined spatial locations in pre- and post-synaptic neurons. One to ten synapses will be spatially located within a 10-μm area and can be analyzed using optical imaging or multisite electrophysiology [planar electrodes of microelectrode arrays (MEAs)]. Thus, this geometrical approach can be further expanded into 3D scaffold construction using biodegradable polymers (Figures 2A,B). To maximize connectivity efficiency, various numbers of channels with asymmetric designs will be tested.

A similar approach was applied to engineer complex neuronal circuits, or even specific brain regions, using microchannels to isolate and guide axon growth between separate networks (Neto et al., 2016; Mobini et al., 2018; Fantuzzo et al., 2019). For example, several groups are modeling complex biologically inspired architecture of hippocampal DG-CA1, CA1-CA3, and CA3-DG regions (Brewer et al., 2013), as well as grafted neurons, in host networks (Shimba et al., 2015).

Furthermore, it is important to optimize neural induction methods to achieve an appropriate excitatory/inhibitory balance when using rodent (and then human) iPS cells. When iPS cells are induced to differentiate into neurons, excitatory or inhibitory neurons are selectively generated because the microenvironment of these cell types is different (Shimba et al., 2019). Thus, to generate a neural population with an appropriate excitatory/inhibitory balance, these neurons need to be generated separately and then mixed at specific ratios. This method can be further optimized to generate a neural population similar to the target tissue (Iida et al., 2018). Differentiated and mixed neurons will be cultured on MEAs to confirm their maturation into functional cells and networks. Next, co-cultured excitatory–inhibitory neurons will be grown using PDMS chips combined with MEAs to confirm that they can form synaptic connections. Such method also permits monitoring of network topology evolution and the functionality of the created network. Conventionally, bioelectrical activity of *in vitro* neural networks significantly differ from *in vivo* conditions. During and after development, hippocampal and cortical networks *in vitro* generate synchronized bursting activity (Wagenaar et al., 2005; le Feber et al., 2010; Pimashkin et al., 2013), which consists of short intervals (hundreds of milliseconds) of high-frequency spiking with long interburting silence (seconds), in contrast to rhythmic and irregular activity in developed networks of the brain. In mature stages of highly dense cultures, spiking activity consists of complex sequences of bursts, often called superbursts, with durations ranging from several to tens of seconds (Wagenaar et al., 2006a, b; Kim et al., 2014; Gladkov et al., 2018). Superburst activity is associated with epileptic seizures *in vitro* (Bao and Wu, 2003). However, recent studies demonstrated that dissociated cultured networks are capable of generating spontaneous activity with *in vivo*-like dynamics under certain conditions. In particular, neuronal cultures were shown to be capable of generating self-replicating spatiotemporal activity patterns (Chiappalone et al., 2007; Schrader et al., 2008; Shahaf et al., 2008; Pimashkin et al., 2011) and exhibit intrinsic mechanisms of synaptic plasticity by adaptation to low-frequency electrical stimulation and training (Shahaf and Marom, 2001; le Feber et al., 2010; Pimashkin et al., 2013). Theta-rhythmic activity can be spontaneously developed in homogeneous cultures of high-density hippocampal cells (Gladkov et al., 2018) or can be induced by inhibitory synaptic transmission modulators on the edge areas of cortical cultures (Keren and Marom, 2016). Similar theta oscillations were observed in septo-hippocampal co-cultures (Fischer et al., 1999). Moreover, 3D cultures grown on MEAs demonstrated *in vivo*-like spiking activity (Frega et al., 2014). Thus, these dissociated culture studies demonstrate the potential for inducing oscillatory *in vivo*-like dynamics using various approaches: use of modulators with particular receptor dynamics, increase of the density and size of the culture, and co-culture of different neuron types. Moreover, the excitatory–inhibitory balance is one of the key parameters responsible for generating stable and reproducible synchronized activity in networks (Eytan and Marom, 2006; Keren and Marom, 2016; Iida et al., 2018). Thus, precise control of the cell type and network connectivity is a key method for engineering a neural circuit for functional integration within the brain.

Microfluidic chips combined with MEAs permit monitoring of electrophysiological signal propagation between chambers (Figure 2D). Note that high-density cultures of approximately 15,000 ± 20,000 cells/mm^2^ with four to five layers of cells that are closer to *in vivo* conditions may also induce rhythmic activity (Gladkov et al., 2018). In such multichamber microfluidic devices, one can mimic neurotransplantation and implant integration using an already developed network. One can grow cultured networks in several chambers, and after 1–2 weeks of maturation, other neurons can be plated into initially free chambers to study how they form connections with previously developed networks and how they influence the integral electrical activity of the culture. Various types of cells can be tested to examine such integration.

## Conclusion

In conclusion, key components of neural circuit formation can be controlled and used to create artificial brain regions on chips with pre-defined synaptic architecture. This model network can be assessed by optical, chemical, and electrophysiological monitoring or stimulation to analyze its functionality. Proposed methods and experimental results can be further used to develop new types of functional scaffolds with the biologically inspired cellular network architecture of iPS cells that can be implemented in 3D structure and tested on rodents for the ability to regenerate or recover in response to brain lesions. Our next step is to present the design of such a 3D structure for cortical lesion recovery, permitting integration of iPS cells or primary neurons into the scaffold to generate a multi-layered network that, after transplantation into the brain, will form synaptic connectivity with the “target” network. The direction of such “repaired” connectivity can be controlled during the implantation stage. Given the comprehensive results of human and mammalian brain connectome analysis, it is now possible to use morphological and network topography anatomy of any region to precisely design and implant neural circuits (Sporns et al., 2005; Bock et al., 2011; Alivisatos et al., 2013; Helmstaedter et al., 2013).

We believe that such methods will significantly improve current neurotransplantation methodology using iPS cells directly from patients. These results can also be used to model any brain region or circuit and used as a transplant in the brain for the fundamental understanding of brain function.

Note that the physical shape of the microchannel design defines the direction of neurite growth and, hence, synaptic architecture of the network. To date, along with vascularization, the lack of innervation of the developed tissue remains a fundamental barrier to engineering full-fledged organs or neuronal scaffolds. Such neural growth control provides innervation, which is required for the development and normal functioning of any type of tissue implanted into the brain. In other words, the proposed technique will elevate the “organ-on-chip” approach and transplantation to a new level. In the field neurodegenerative diseases, a number of CNS diseases are attributed to alterations in neuronal circuitry (schizophrenia, Alzheimer’s disease, etc.). This presents the possibility of a proposed hybrid system that would permit direct implementation of designed circuitry to model networks with particular diseases to uncover this circuit imbalance.

## Data Availability

All datasets generated for this study are included in the manuscript and/or the supplementary files.

## Ethics Statement

The protocol was approved by the Committee on the Ethics of Animal Experiments of the Nizhny Novgorod State Medical Academy (Permit Number: 9±25.09.2014).

## Author Contributions

KS, C-HC, TA, FM, AG, OA, YP, and VlK performed the preliminary experiments. AP, ViK, IM, KS, and C-HC wrote the manuscript in consultation with KK and YJ. All authors contributed to the final manuscript.

## Conflict of Interest Statement

The authors declare that the research was conducted in the absence of any commercial or financial relationships that could be construed as a potential conflict of interest.
